# Analysis of chicken and pig DNA content in commercial dry foods for adult cats

**DOI:** 10.1007/s11259-024-10513-x

**Published:** 2024-08-23

**Authors:** Jagoda Kępińska-Pacelik, Wioletta Biel, Małgorzata Natonek-Wiśniewska, Piotr Krzyścin

**Affiliations:** 1 Department of Monogastric Animal Sciences, Division of Animal Nutrition and Food, West Pomeranian University of Technology in Szczecin, Klemensa Janickiego 29, Szczecin, 71-270 Poland; 2 Department of Animal Molecular Biology, National Research Institute of Animal Production, Krakowska 1, Balice, 32-083 Poland

**Keywords:** Mislabeling, Cats, Extrusion, Food allergy, Food fraud, Real-time PCR

## Abstract

Among pets, cats are the most popular in Europe. Despite the fact, the interest in the safety and quality of their food is much lower compared to the interest of caregivers in the nutrition of dogs. In this research, 27 commercial cat foods were analyzed for mislabeled component composition. Cat foods were divided into a control group, a group of fish foods and a group of other foods with alternative sources of animal protein. Chicken and pig DNA detection was performed using real-time PCR. In this research, 100% of the cat foods contained chicken DNA and 96% of the foods – pig DNA, despite the lack of declaration of these ingredients on the product label. The results indicate that cat food appear to be mislabeled to an even greater extent than dog food. Moreover, manufacturers’ declarations in terms of ingredient composition do not reflect the actual composition of commercial products available on the market and intended for everyday feeding of animals. Mislabeling of these products also poses a risk for animals suffering from food allergies.

**Supplementary Information:**

The online version contains supplementary material available at 10.1007/s11259-024-10513-x.

## Introduction

The domestic cat (*Felis catus* L.) is currently the most popular companion animal in Europe. According to the Facts and Figures Report published by the European Pet Food Industry Federation (FEDIAF) (2023), the number of households in which cats live is estimated at 91 million. Cats have coexisted with humans for thousands of years, and their main role in households was to catch pests which destroyed food supplies (Driscoll et al. 2009). Cats are obligate carnivores - in the wild they are mainly nocturnal. In the case of indoor cats that are dependent on a caregiver, they are relatively often fed commercial dry or wet food (Biró et al. 2005; Doherty et al. 2015).

One of the most frequently diagnosed diseases in both dogs and cats is food allergy. A common way to diagnose the food allergy is to identify the ingredient in the pet food causing allergic reaction (Bryan and Frank 2010). If a food allergy or hypersensitivity to a specific protein is suspected, an elimination diet, and then the provocation tests, are often performed. They involve administering a potentially allergenic protein to the animal in order to induce an allergic reaction. What is important, an elimination diet can only be performed on indoor cats. Caregivers often feed cats with food allergy with commercial food on their own. In the case of elimination diet and provocative tests, the fact that cat food is mislabeled with a protein other than that declared by the manufacturer may occur problematic (Pulina et al. 2021). Currently, in the food industry, the most frequently consumed species of slaughter animals in European Union (EU) are chicken and domestic pig (Eurostat 2023). Therefore, the largest amounts of animal by-products are of these species, and consequently they are cheaper than more unusual species (Preckel et al. 2021). Therefore, food ingredients can be expected to be mislabeled with these species of slaughter animals due to economic reasons and imprecise legal regulations. Commission Regulation No. 767/2009 allows the list of components by categories in pet foods (Regulation 767/2009).

In the case of dogs, the scientific literature is rich in terms of the latest scientific research focusing on their nutrition, safety and quality of dog food (Dunham-Cheatham et al. 2021; Kazimierska et al. 2020; Kazimierska et al. 2021; Kępińska-Pacelik et al. 2023a; Kępińska-Pacelik et al. 2023b). Unfortunately, the same cannot be said about cats. Although the great popularity of cats in Europe, there are few recent scientific publications regarding their nutrition. Furthermore, the safety and quality of food intended for cats are still marginally treated. Therefore, the aim of the study was to analyze the DNA content of chicken (*Gallus gallus domesticus*) and pig (*Sus domestica*) in 27 commercial complete extruded foods for adult cats.

## Materials and methods

### Material

From the base of complete commercial extruded dry cat foods that do not contain pig protein and fat, 27 products were selected in packages in the range of 0.3–2.5 kg. These foods were bought at pet stores and online stores in January 2023. All bags were stored in laboratory (temperature 18–21 °C) until analysis. From each bag, a representative sample was collected for laboratory analyses (ISO 2012). The analyzed foods were homogenized in sterile conditions, placed in sterile containers and marked with the symbols C1–C27 (Table 1). Then representative samples were prepared from which three laboratory samples of 100 mg were weighed.

### DNA identification

Total DNA from each laboratory sample was obtained using the AX Food kit (A&A Biotechnology, Gdynia, Poland), in accordance with the methodology of the producer’s recommendations and finally suspending the DNA isolate in 50 µl of TE buffer. DNA concentration and purity were measured using a NanoDrop photometer spectrometer (ND 2000, NanoDrop Technologies, Wilmington, DE, USA). Purity was determined on the basis of the absorbance ratio at wavelengths 260 and 280. The quality of the obtained DNA isolations was determined on the basis of the extraction repeatability coefficient (Wr) and precision of the concentrations (R) and purity (P). Repeatability coefficient was describe by comparing the mass of the each analyzed sample and the obtained DNA concentration from it. Whereas the precision of the obtained concentrations or purity were determined on the basis of the relative difference between the highest and lowest concentration / purity (A_260/280_). Formulas determining the numerical value of the above quantities are presented in Supplemental File.

Determination of the amount of chicken and pig components was carried out in a thermal cycler Step One Plus™ Real-Time PCR v2.3 (Thermo Fisher Scientific, MA, USA). Primers (Genomed SA, Warsaw, Poland) and probes TaqMan^®^MGB (Thermo Fisher Scientific, MA, USA) were homologous to fragment 16 of small regulatory RNA (srRNA) and cytochrome c oxidase subunit I (COX1) for chicken and pig respectively (Natonek-Wiśniewska and Krzyścin 2016). The thermal program included 40 cycles and its annealing temperature was 60 °C. To increase the accuracy of the study, the analyzes were performed in repetitions from three independent DNA isolations. Additionally, to confirm the accuracy of the analyses, a DNA isolation and PCR positive control (PC), a DNA isolation negative control (NC), and a PCR negative control (NTC) were performed. Positive controls were a meat sample containing 0.5% of meat from the species being determined, while negative controls were a water sample. Amplification thresholds cycles (Cт) were determined for all samples, and then the amount of DNA was calculated based on a standard curve of five dilutions of 0.01–100% (factor = 10) of DNA obtained from a mixture of a single species template of the designated species. All calculations were performed in a quantitative manner using the polymerase chain reaction (qPCR) monitoring program, which is an integral part of the thermal cycler. The standard curve was assessed based on its parameters: shift, slope and regression coefficient (R) (Biel et al. 2022). Concentration is presented as the mean of three measurements for the same sample, the standard deviation (SD%) between them, and the relative standard deviation (RSD%). These parameters were calculated using the Eqs. (1) and (2):

1 $$\:\text{S}\text{D}=\sqrt{\frac{\sum\:_{\text{i}=1}^{\text{n}}({\stackrel{-}{\text{c}}-\text{c})}^{2}}{\text{n}-1}}$$

2 $$\:\text{R}\text{S}\text{D}=\frac{\text{S}\text{D}}{\stackrel{-}{\text{c}}}\:\times\:100$$

Moreover, for positive samples the accuracy was calculated according to the Eq. (3):

$$\:\text{A}\text{C}\%=\frac{\text{c}\:\text{c}\text{a}\text{l}\:-\:\text{c}\:\text{a}\text{c}\text{t}}{\text{c}\:\text{a}\text{c}\text{t}}$$ × 100% (3)

where:

c cal – calculated value of percentage concentration;

c act – actual value of percentage concentration.

## Results

This research has shown that the problem of mislabeling the composition of cat food is sorely serious – 100% contained chicken DNA and 96% pig DNA, although the manufacturer did not declare the presence of these ingredients on the label (Table 1).

Table 1 summarizes the main ingredients of the tested foods. Out of 27 analyzed cat foods, 3 groups were distinguished;

1. a group of foods with chicken as a control group (C1–C10),
2. a group of foods with fish (C11–C20),
3. a group of foods with alternative sources of animal protein (C21–C27).

As expected, the presence of chicken was confirmed in all foods from group 1. However, pig DNA was also detected in all foods from this group, even though its presence was not declared by the manufacturer. In group 2, in which foods contained only fish, both chicken and pig DNA were identified. Interestingly, C18 was the only food in this research in which pig DNA was not detected. Group 3 was characterized by the presence of both chicken and pig DNA, although the manufacturers declared only alternative sources of animal protein, such as rabbit, lamb, goat, insects.

**Table 1:** Main animal components of tested foods and identified species

Item	Manufacturer’s declaration		Identified species (G or S)
	Animal components	Animal fat source	
C1	chicken meat, eggs	salmon oil	G, S
C2	poultry protein, eggs	poultry fat, salmon oil	G, S
C3	chicken meat	chicken fat, fish oil	G, S
C4	chicken meat, poultry protein, eggs	animal fat, fish oil	G, S
C5	chicken meat, chicken protein, eggs, herring protein	chicken fat, herring oil	G, S
C6	poultry meat powder, greaves	poultry fat, beef fat	G, S
C7	poultry protein, animal protein	animal fat, fish oil	G, S
C8	chicken protein, animal protein	purified animal fat	G, S
C9	poultry protein, greaves, lamb protein, hemoglobin, fish, eggs, mussel meat	animal fat (including poultry), sunflower oil	G, S
C10	chicken, deer, salmon, fish	chicken fat	G, S
C11	carp fish, herring meat, salmon, fish protein	salmon oil	G, S
C12	salmon, salmon protein	salmon oil	G, S
C13	trout, fish meal, salmon	NDL	G, S
C14	salmon, salmon protein, white fish protein, sardine protein	salmon oil	G, S
C15	herring, mackerel, hake, blue whiting, flounder, redfish, cod liver	pollack oil	G, S
C16	salmon, herring, hake, rainbow trout	pollack oil, salmon oil	G, S
C17	herring, insects	herring oil	G, S
C18	sardine, hake, mackerel, flounder, redfish, sole, blue whiting, whitefish	pollack oil	G
C19	fish, salmon meal, fish meal	NDL	G, S
C20	fish	salmon oil	G, S
C21	rabbit	salmon oil	G, S
C22	offal and lamb meat	NDL	G, S
C23	beef	beef fat	G, S
C24	goat meat, wild boar meat, deer meat, Arctic char, duck meat, mutton, rainbow trout	duck fat, herring oil	G, S
C25	beef, wild boar, buffalo, lamb	beef fat, herring oil	G, S
C26	insect protein	salmon oil	G, S
C27	Hermetia illucens larvae	insect oil	G, S

*G – Gallus gallus domesticus*, *S – Sus domestica*, *NDL –* Not declared on the label

The DNA isolation method used in these analyses allowed to obtain solutions with concentrations ranging from 59.207 to 229.768 ng/µl (Table 2). The working range of reproducibility of the samples between independent DNA isolations on the same food shows a difference of less than 35% for majority of them (Table 2). A low range of reproducibility was observed only for two samples: C13 and C26, for which this value is below 50%. Similarly, the precision of the obtained concentrations was most often above 70% (Table 2), although there were samples for which the difference was higher and was even around 65% in relation to the maximum concentration for a given sample (C13, C22). The purity of the concentrations ranged the most often from 1.60 to 1.81, and only in few cases the purity was below this value, additional not in all replicate of samples. This can be directly translated into the precision of this measurement, which was mostly below 5%, and only for one sample exceeded 10%. Despite the DNA extraction values for some replicates are not the best, they were sufficient for further analysis for all isolates.

**Table 2:** Parameters of the obtained DNA isolates

Sample name	c ng/ul			A_260/280_			Wr%			*R*%	*P*%
							min	max			
C1	114.116	144.646	131.940	1.70	1.75	1.72	79.67		107.50	21.11	2.86
C2	92.653	130.136	117.665	1.72	1.76	1.74	76.89		103.43	28.80	2.27
C3	194.029	144.487	229.768	1.81	1.76	1.79	69.24		121.97	37.12	2.76
C4	155.798	121.357	166.893	1.76	1.73	1.74	79.33		118.78	27.28	1.70
C5	194.974	158.103	192.948	1.77	1.76	1.78	81.13		119.76	18.91	1.12
C6	138.034	147.875	152.121	1.62	1.74	1.74	85.64		102.07	9.26	6.90
C7	134.893	136.803	147.522	1.73	1.73	1.69	91.44		97.66	8.56	2.31
C8	139.800	152.014	185.415	1.73	1.77	1.76	75.40		94.67	24.60	2.26
C9	108.432	107.933	89.488	1.69	1.73	1.73	101.45		118.79	17.47	2.31
C10	119.707	130.700	114.394	1.57	1.73	1.73	95.25		115.35	12.48	9.25
C11	125.858	103.690	93.910	1.70	1.71	1.70	111.48		134.02	25.38	0.58
C12	94.761	109.892	66.592	1.69	1.73	1.71	82.95		165.02	39.40	2.31
C13	51.011	143.731	81.520	1.45	1.78	1.73	35.85		183.30	64.51	18.54
C14	59.459	49.213	51.202	1.69	1.74	1.69	91.54		120.82	17.23	2.87
C15	90.596	80.122	75.074	1.72	1.70	1.66	108.80		123.02	17.13	3.49
C16	161.161	151.481	148.455	1.74	1.71	1.75	103.04		107.51	7.88	2.29
C17	124.997	92.817	93.745	1.74	1.69	1.65	97.12		135.97	25.74	5.17
C18	136.853	164.900	138.349	1.72	1.75	1.73	79.83		121.55	17.01	1.71
C19	144.245	104.401	158.573	1.74	1.71	1.77	63.92		142.31	34.16	3.39
C20	144.822	96.521	126.660	1.74	1.70	1.73	80.02		137.65	33.35	2.30
C21	119.945	90.092	116.751	1.81	1.73	1.81	76.40		128.06	24.89	4.42
C22	15.207	22.106	39.613	1.67	1.55	1.59	42.27		70.88	61.61	7.19
C23	68.383	113.558	100.554	1.70	1.72	1.70	62.60		108.63	39.78	1.16
C24	159.126	143.491	135.336	1.77	1.74	1.62	101.99		118.75	14.95	8.47
C25	23.157	30.434	21.228	1.57	1.71	1.60	77.57		143.37	30.25	8.19
C26	112.919	57.470	110.661	1.75	1.60	1.70	50.94		200.30	49.11	8.57
C27	54.584	44.834	71.542	1.59	1.66	1.65	68.31		119.36	37.33	4.22

c – DNA concentration, A_260/280_ – ratio absorption of DNA, Wr% – working repeatability range described in percentage, R% – precision of concentration described in percentage, P% – precision of purity described in percentage

**Table 3:** Concentrations of chicken DNA in analyzed cat foods

Sample name	Target name	c_T_ mean	c_T_ SD	Mean quantity(g/kg)	Quantity SD
C1	Gall	15.2978	0.0643	276.75	12.38
C2	Gall	15.7822	0.2170	198.87	30.92
C3	Gall	15.0422	0.3660	337.48	81.62
C4	Gall	15.4257	0.1349	253.55	16.84
C5	Gall	16.1136	0.0544	156.71	6.01
C6	Gall	17.4625	0.0733	61.23	3.09
C7	Gall	16.2573	0.1140	142.01	11.23
C8	Gall	16.4008	0.1022	128.44	9.22
C9	Gall	19.3006	0.1994	17.10	2.27
C10	Gall	14.7543	0.4173	415.09	114.95
C11	Gall	21.4825	0.4403	3.77	0.81
C12	Gall	22.1549	0.2264	2.34	0.38
C13	Gall	17.5229	0.2364	59.19	9.60
C14	Gall	17.1031	0.0596	78.64	2.31
C15	Gall	26.6969	0.4421	0.10	0.03
C16	Gall	25.0775	0.1836	0.30	0.04
C17	Gall	17.8724	0.0412	45.99	1.31
C18	Gall	25.4884	0.6109	0.24	0.10
C19	Gall	21.0158	0.1832	5.17	0.65
C20	Gall	24.9497	0.3091	0.34	0.07
C21	Gall	19.7282	0.3148	12.83	2.96
C22	Gall	22.8407	0.2059	1.43	0.15
C23	Gall	22.0017	0.2045	2.60	0.36
C24	Gall	23.0290	0.0433	1.26	0.04
C25	Gall	24.9069	0.4888	0.48	0.14
C26	Gall	21.3896	0.1146	5.11	0.40
C27	Gall	21.2687	0.2355	5.58	0.92
NC	Gall	UND		NR	NR
PC 0.5%	Gall	21.2186	0.0518	4.47	0.16
NTC	Gall	UND		NR	NR

*Gall – Gallus gallus domesticus* target, *UN*D – Undetermined, *NR –* No reaction, *NTC –* Negative control of PCR, *NC –* Negative control of DNA isolation, *PC 0.5% –* Positive control of DNA isolation and PCR, *c*_T_*mean *– mean value of threshold cycle, *c*_T_*SD –* standard deviation of threshold cycle, *Quantity SD –* standard deviation for the quantitative results obtained

In the group of foods with chicken as the only source of animal protein, the mean quantity of chicken DNA ranged from 17.10 in food C9 to 415.09 in food C10 (Table 3). Although the producers did not declare the presence of chicken in any of the C11–C20 cat foods with fish, mean quantity of chicken DNA were detected in all of these foods in the range of 0.10 in food C15 to 78.64 in food C14. Similarly, in the group of foods with other animal sources, the presence of chicken DNA was detected in all of these foods, in the range from 0.48 in food C25 to 12.83 in food C21. The food C25 according to the producer’s declaration contained beef, wild boar, buffalo, and lamb. In case of the food C21, the animal component, according to the declaration, was rabbit. It is worth noting that in three foods (C15, C18, C25) the content of chicken DNA was below the limit of quantification (LOQ), which may indicate only trace contamination of these foods due to cross-contamination.

**Table 4:** Concentrations of pig DNA in analyzed cat foods

Sample name	Target name	c_T_ mean	c_T_ SD	Mean quantity(g/kg)	Quantity SD
C1	Sus	24.3821	0.0812	4.37	0.23
C2	Sus	22.1411	0.3437	18.87	4.17
C3	Sus	31.2447	0.3383	0.05	0.01
C4	Sus	20.7852	0.3296	45.21	9.00
C5	Sus	21.8394	0.1604	22.64	2.40
C6	Sus	18.6467	0.2442	178.69	26.88
C7	Sus	27.1134	0.3228	0.76	0.17
C8	Sus	27.7955	0.2485	0.49	0.08
C9	Sus	18.8668	0.1042	154.01	10.31
C10	Sus	24.6412	0.1664	3.71	0.39
C11	Sus	20.9662	0.0495	39.66	1.27
C12	Sus	31.6940	0.0450	0.04	0.00
C13	Sus	19.8496	0.5179	81.77	6.36
C14	Sus	26.8127	0.2047	0.91	0.12
C15	Sus	31.4735	0.5545	0.05	0.02
C16	Sus	34.3645	0.3898	0.01	0.00
C17	Sus	24.8360	0.1648	3.27	0.34
C18	Sus	36.6097	2.8589	0.00	0.00
C19	Sus	34.1946	0.0740	0.01	0.00
C20	Sus	30.8763	0.6370	0.07	0.03
C21	Sus	20.5421	0.2468	52.57	8.23
C22	Sus	34.1160	0.1598	0.01	0.00
C23	Sus	24.2136	0.1797	4.89	0.55
C24	Sus	24.3530	0.3610	4.53	1.00
C25	Sus	18.5705	0.1127	118.35	8.56
C26	Sus	23.6714	0.5006	4.66	1.34
C27	Sus	29.6172	0.2207	0.10	0.01
NC	Sus	UND		NR	
PC 0.5% 11 A/16	Sus	24.5374	0.0740	3.95	0.19
NTC	Sus	UND		NR	

*Sus – Sus domestica target*, *UND –* Undetermined, *NR –* No reaction, *NTC –* Negative control of PCR, *NC –* Negative control of DNA isolation, *PC 0.5% sus* – Positive control of DNA isolation and PCR, *c*_T_*mean –* mean value of threshold cycle, *c*_T_*SD –* standard deviation of threshold cycle, *Quantity SD –* standard deviation for the quantitative results obtained

Most cat foods contained pig DNA ranging from 0.01 to 178.69, except one - C18. A high content of pig DNA was found mainly in the group of foods in which the animal component declared by the manufacturer was only chicken, especially in the C6 and C9 foods. In the group of foods with fish, the values obtained in the C13 food (81.77) attract particular attention, in which the animal components declared by the manufacturer were trout, fish meal and salmon. Similarly, the mean quantity of pig DNA 39.66 in C11 food, the animal ingredients of which, according to the manufacturer’s declaration, were carp fish, herring meat, salmon, and fish protein, may cause concern. In the group of other foods, particular attention is paid to the pig DNA content of 118.35 in the C25 food, in which the ingredients declared by the manufacturer were beef, wild boar, buffalo, and lamb. What is important, in the three analyzed foods (C16, C19, C22) the pork product content was below the LOQ, which indicates only a trace amount of pig DNA in these foods.

## Discussion

As reported in the Facts and Figures Report published by FEDIAF (2023), 91 million households in the EU own a pet, and cats are the most popular species. The annual growth rate for the pet food industry in 2022 was near 5%. Moreover, 10.5 million tons of pet food products were sold annually, and profits from the sale of pet food products amounted to 29 billion euro.

In previous years, the most popular option was to feed cats with commercial food available on the market. In the study by Dodd et al. (2020), 90% of cats received commercial food. Cats are obligate carnivores, therefore their diet should consist exclusively of animal products. In the case of commercial dry foods, this involves large financial outlays to obtain meat raw materials, which are more expensive than plant raw materials. The production process also requires the use of texturizing raw materials– plants. Mislabeling of pet food by manufacturers is observed for economic gain at the expense of the animal’s health, as we noted in our previous studies on mislabeling of dog food (Biel et al. 2022; Kępińska-Pacelik et al. 2023a). As shown by Nesvadbova et al. (2022), analysis of retail pet food samples showed the presence of undeclared species in 60% of the samples.

Moreover, in the fifteen years (from 2007 to 2022), meat production has increased, especially for chicken and pigs, for which production has increased by 11.5 and 4.3 times, respectively, over 50 years (Gilbert et al. 2015). Chicken and pig are cheaper meat species and are used as a substitute for species with a higher economic value (Vatin et al. 2023). Economic considerations may lead to the frequent detection of these animal species as mislabeling components in cat food.

Food producers and sellers are obliged to provide consumers with correct information about food (Cichna-Markl and Mafra 2023). However, despite the fact that food manufacturers are bound by the Code of Good Labelling Practice for Pet Food (FEDIAF 2019), food labels often contain false or misleading statements regarding the composition, quality, geographical origin and/or processing of food. However, due to publicized safety problems with pet products, public trust in manufacturers has decreased (Dunham-Cheatham et al. 2019). In the era of widespread use of food mislabeling practices, food authentication is a major challenge and requires highly selective, sensitive, accurate, repeatable analytical methods (Feltes et al. 2023). Due to these parameters, the technique most often chosen for this type of research is real-time PCR (Natonek-Wiśniewska et al. 2022a). This also applies to species authentication in meat products, for which real-time PCR is one of the most commonly used DNA-based techniques (Natonek-Wiśniewska et al. 2022b; Cichna-Markl and Mafra 2023; Kusnadi et al. 2023).

Several assays have been developed that utilize next generation sequencing technology. One of them is the next-generation semiconductor-based sequencing technology (Ion Torrent Personal Genome Machine). Bertolini et al. (2015) used this technology to identify DNA of meat species (pig, horse, cattle, sheep, rabbit, chicken, turkey, pheasant, duck, goose) and pigeon), as well as human and rat DNA in DNA mixtures by sequencing PCR products obtained from different pairs of universal primers that amplify the 12 S and 16 S rRNA mitochondrial DNA genes. The error rate calculated after confirming the obtained sequences by Sanger sequencing ranged from 0.0003 to 0.02 for different species. Detection of low levels of DNA was possible with reads obtained from different primer pairs, and sequencing the products obtained from different universal PCR primers may be a useful strategy to overcome potential amplification problems (Bertolini et al. 2015).

Kattoor et al. (2024) developed a targeted next-generation sequencing panel for detecting meat species in canned pet food using Ion Torrent technology. This panel contains multiple primers targeting mitochondrial genes for as many as 27 animal species, of which 7 major animal species have been validated. Target meat species can be identified from samples spiked with as little as 0.01% of the contaminating meat species in the vegetarian food matrix material, making this panel a very sensitive method.

Among the available mislabeling detection techniques, DNA barcoding is one of the ones with the greatest advantages. It is based on the principle of using standardized, unique DNA sequences of 400–800 bp in length, mitochondrial (e.g. COI) or plastidial (e.g. rbcL) of nuclear origin (e.g. ITS) for the analysis and classification of food commodities. Each commercially traded product, such as legumes, seafood, oils, herbal products, spices, fruit, grains and meat, has unique barcodes that can be analyzed to detect misabeing or fraud (Nehal et al. 2021).

Some of the earliest reported cases of human food fraud, dating back thousands of years, involved olive oil, tea, wine and spices. These products, like some other food products, continue to be associated with fraud. Unfortunately, adulterations and mislabeling are increasingly common in products intended for pets (Kępińska-Pacelik et al. 2023a). Zhu et al. (2023) detected undeclared animal species in all 6 of the tested pet foods. Foods intended to contain lamb contained beef and venison. And the foods that were supposed to contain beef, were mislabeled with game. Although the proportions of bovine DNA found in the samples were low (< 1%), the proportions of venison DNA were over 3.5%. These results indicate the partial replacement or contamination of lamb with venison and/or beef in pet food. Okuma and Hellberg (2015) analyzed 52 commercial pet products for the presence of eight meat species (cattle, goats, sheep, chickens, geese, turkeys, pigs and horses) using real-time polymerase chain reaction. Among the products tested, 31 were correctly labeled, 20 were potentially mislabeled, and 1 contained a non-specific meat ingredient that could not be verified. The pet foods were most often mislabeled with chicken (51 out of 52). Dunham-Cheatham et al. (2021) reached similar conclusions – ingredients with higher economic value are often supplemented or completely replaced with these of lower value, e.g. chicken. An example is the case of mono-protein food, in which the manufacturer declared tuna and salmon as animal ingredients, although research has shown that the animal ingredients are dominated by chicken, sheep and turkey (Dunham-Cheatham et al. 2021). Moreover, raw meat diets are becoming popular these days. However, cat caregivers cannot be sure of the reliability of their composition too. As shown by Cox et al. (2020), turkey was most frequently detected in raw diets for cats.

Maine et al. (2015) analyzed the presence of undeclared sources of animal protein in 17 pet foods. Most samples detected significant numbers of unspecified animal species. Bovine, pig and chicken DNA was identified in 82% of samples (14 out of 17) in various proportions and combinations, however these species were not listed on product labels. Of the 7 products claimed to contain beef, only 2 contained more beef DNA (> 50%) than pig and chicken DNA combined. Similarly, in the study of Palumbo et al. (2020), 89% of pet foods (16 out of 18) were found to be mislabeled. Given these results and subsequent studies, there may be concerns that consumers are paying unfair prices for products that claim to contain high-value ingredients but actually contain low-value ingredients. The incidence of mislabeling in commercial pet foods is also a problem for pets suffering from life-threatening food allergies.

The issue of food allergies and intolerances in companion animals is problematic. It has been observed in clinical veterinary medicine for many years that the incidence of adverse food reactions (AFR) is correlated with the dynamic development of the pet food industry. Lack of optimization of the composition in terms of nutrient content, identified deficiencies and excesses of individual minerals and, above all, the presence of undeclared ingredients constitute a constant immunological and cellular challenge for the animal’s body (Dodds 2019). The diagnosis of AFR in pets is mainly based on the results of provocation tests, which involve giving the animal potentially allergenic food in order to observe whether an allergic reaction occurs. For allergenic ingredients in cats’ diets, the most frequently reported ingredients were beef, fish, chicken, lamb, wheat, corn and dairy products. Some cats have also shown allergic reactions to eggs, barley and rabbit (Guilford et al. 2001; Guaguère et al. 1993). However, the data obtained do not allow estimating the actual occurrence of allergens in the cat population because the animals were usually challenged with a small number – but not all – of the allergens. As a result, the actual prevalence of each allergen in cats is likely to be higher due to the potential for synergistic effects of allergens (Mueller et al. 2016). Despite the popularity of cats as pets, little is known about the pathogenesis of AFR in them. The vast majority of information focuses on knowledge about humans and dogs. Allergic reactions in cats result from an excessive immune response to antigens, mainly causing diarrhea, itching and skin lesions (Mueller and Unterer 2018; Colombo 2020). Itching in cats can manifest itself in various forms, including: by scratching, licking, biting the fur or shaking the head in case of itchy ears. Scratching in cats is part of normal behavior, but when it is excessive and lesions occur at the same time, the caregiver can easily recognize it. In cats, AFR is associated with gastrointestinal symptoms in almost 20% of cases, such as vomiting, flatulence, soft stools, increased defecation or diarrhea (Hobi et al. 2011). If diseases causing disturbing symptoms have been ruled out and the symptoms still persist, an elimination diet should be introduced to confirm or rule out AFR.

According to the current state of knowledge, no serological test has proven reliable in the diagnosis of AFR. However, according to the findings from the study of Noli and Beltrando (2021), diagnosis of AFR in cats can be successfully performed using the commercially available hydrolyzed fish protein and rice). In that study, 17 of 35 (49%) cats had a > 50% reduction in pruritus and the cats were treated with dietary therapy. Nine of them responded to the previous diet and/or fish and/or rice and were diagnosed with AFR, while eight cats did not relapse.

Nevertheless, an elimination diet and subsequent challenge tests are the only treatment usually recommended by veterinarians. The elimination diet should last for at least 8 weeks (Noli and Beltrando 2021). Unfortunately, many caregivers use food that does not contain any potentially allergenic ingredients as an elimination diet on their own. Due to the high percentage of mislabeled foods, there is a risk of false results because the cat will react to the food even though theoretically it should not cause an allergic reaction according to the manufacturer’s declaration (Colombo 2020).

It is worth noting that mislabeling a product with a foreign protein does not always have to be a deliberate attempt by the manufacturer. Also, it has been shown that undeclared animal species can be as common as the lack of animal protein declared on the label (Biel et al. 2022). In pet food factories, where different types of raw materials pass through production lines, there is a risk of cross-contamination of the product. According to research by Chung and Hellberg (2019), cross-contamination can be avoided provided that the equipment is thoroughly cleaned between each production batch in accordance with the instructions and recommendations. However, if procedures are not followed, product contamination is highly likely to be observed, with levels of < 1% of undeclared species. Nevertheless, whether intentional or not, mislabeling is a threat to animals suffering from food allergies and unfair behavior towards cat caregivers who expect the manufacturer’s declaration to be consistent with the actual composition of the food.

Over the years, the presence of undeclared DNA in pet food has been analyzed, but most research focuses on food for dogs. The safety of food for cats is still treated marginally, and as this research has shown, it is mislabeled to an even greater extent than is observed in dog food. In this research, 100% of the foods contained chicken DNA and 96% pig DNA. These days the awareness of animal caregivers about proper nutrition is increasing, but understanding the essence of nutrition will not help much as long as producers use unfair practices when producing pet food, therefore products intended for companion animals should be more transparent. A reliable manufacturer’s declaration on the product label, including all species of animals used to produce the food, will enable caregivers to make a conscious choice when purchasing the pet food. Applying fair practices in the cat food industry will reduce the risk of product misinterpretation by caregivers, which is especially important in the case of animals suffering from food allergies.

## Supplementary Information

Supplementary Material 1

## Data Availability

No datasets were generated or analysed during the current study.

## Abbreviations

AC: Accuracy
AFR: Adverse food reactions
COX1: Cytochrome c oxidase subunit I
C_T_: Threshold cycle
EU: European Union
FEDIAF: European Pet Food Industry Federation
LOQ: The limit of quantification
NC: DNA isolation negative control
NTC: PCR negative control
P: Purity
PC: DNA isolation/PCR positive control
qPCR: quantitative polymerase chain reaction
R: precision of the concentrations
R^2^: Regression coefficient
RSD: Relative standard deviation
SD: Standard deviation
srDNA: small regulatory RNA
Wr: extraction repeatability coefficient
