# Supplementary material for: Analysis of chicken and pig DNA content in commercial dry foods for adult cats
Source: Vet Res Commun. 2024 Aug 23;48(6):4051–9. doi: 10.1007/s11259-024-10513-x (PMC11538195; doi:10.1007/s11259-024-10513-x)
Supplement: Supplementary file 1 — Supplementary Material 1 [file 11259_2024_10513_MOESM1_ESM.docx]

**Veterinary Research Communications**

**Analysis of chicken and pig DNA content in commercial dry foods for adult cats**

Jagoda Kępińska-Pacelik^1*^, Wioletta Biel^1^, Małgorzata Natonek-Wiśniewska^2^, Piotr Krzyścin^2^

^1^Department of Monogastric Animal Sciences, Division of Animal Nutrition and Food, West Pomeranian University of Technology in Szczecin, Klemensa Janickiego 29, 71-270 Szczecin, Poland, orcid.org/0000-0002-3385-6281 (W.B.), orcid.org/0000-0001-5925-0694 (J.K-P.)

^2^Department of Animal Molecular Biology, National Research Institute of Animal Production, Krakowska 1, 32-083 Balice, Poland, orcid.org/0000-0003-2132-2829 (M.N-W.)

^*^corresponding author: e-mail: jagoda.kepinska-pacelik@zut.edu.pl

Wr% = $\frac{c1m2}{c2m1} 100\%$ where:

m1 – mass of the first sample repetition;

c1 – concentration of DNA obtained from the sample;

m2; c2 – the same parameters which were obtained for the next repetition.

R% = $\frac{cmax-cmin}{\mathrm{cmax}} 100\%$

where:

cmax – the higher concentration of DNA obtained from the sample;

cmin – the lower concentration of DNA obtained from the same sample.

P% = $\frac{A260/280 max-A260/280min}{A260/280max} 100\%$

where:

A_260/280_ max – the higher ratio absorption of DNA obtained from the sample;

A_260/280_ min – the lower ratio absorption of DNA obtained from the same sample.
